# Aggressiveness that makes one grow: a destructive or vital force in adolescence? Reflections and working hypotheses

**DOI:** 10.3389/fnbeh.2026.1763952

**Published:** 2026-02-17

**Authors:** Francesco Demaria, Maria Pontillo, Cristina Di Vincenzo, Stefano Vicari

**Affiliations:** 1 Child and Adolescence Neuropsychiatry Unit, Bambino Gesù Children’s Hospital, IRCCS, Rome, Italy; 2 Department of Psychology, Catholic University of the Sacred Heart, Milan, Italy; 3 Department of Life Sciences and Public Health, Catholic University of the Sacred Heart, Rome, Italy

**Keywords:** adolescence, aggression, aggressive behaviors, development, growth

## Abstract

Adolescence is a critical developmental stage marked by profound physical, emotional, and social transformations. During this stage, bodily changes, emotional vulnerability, environmental stimuli, and cultural influences may expose adolescents to behaviors that appear unpredictable or maladaptive. Moreover, a central aspect of adolescence is the drive for self-affirmation, which often manifests through aggression, in actions, thoughts, and interpersonal relationships. More than merely an individual reaction, adolescent aggression also constitutes a broader social phenomenon. The present work seeks to explore the contemporary role played by aggression in adolescence and its varied expressions. It supports a paradigm in which adolescent aggression fluctuates between two poles: one aligned with the pleasure principle, characterized by self-gratifying behavior that disregards others and seeks unmediated satisfaction; and the other aligned with the reality principle, characterized by self-affirming behavior grounded in moral consciousness and respect for social norms. The dominance of either form of aggression plays a crucial role in shaping adolescent development. In contemporary society, cultural models and myths promote a narcissistically driven, empathy-deficient form of aggression. This mode of behavior, which is socially rewarded and normalized, risks becoming the adolescent’s internalized version of the reality principle, with success achieved at the expense of others. Such a framework may inhibit the transformation of aggression into socially and morally attuned behavior. Thus, it is essential to understanding adolescent aggression as a powerful, though ambivalent, force in the human development.

## 1 Introduction

Adolescence represents a critical transition phase prior to adulthood, marked by profound biological and psychological transformation (Rosenfeld and Nicodemus, 2003). Bodily changes, heightened emotional sensitivity, and new social demands converge to create a physiological and psychological crisis that impacts cognitive processes, impulse regulation, and the adolescent’s perception of self and others. The earlier onset of puberty, signaling the beginning of adolescence, and the delayed achievement of traditional adult tasks (e.g., completing education, entering marriage, becoming a parent) are prolonging this developmental stage (Sawyer et al., 2018), renders adolescents increasingly vulnerable to internal conflict, emotional disturbances, and intensified exposure to environmental stimuli and cultural influences (Martin et al., 2014).

In contemporary society, adolescents are compelled to confront and test themselves, idealize external models, and place significant emphasis on their image and appearance, often amplified by a pervasive use of digital technology and social media. These dynamics may impair their relational and emotional intelligence, fostering impatience and discomfort that may heighten their risk of behavioral deviation (Choukas-Bradley et al., 2022; Jarman et al., 2021). Such vulnerability can serve as fertile ground for aggression to emerge as a mechanism of self-affirmation and a potentially vital force in the adolescent’s developmental process. It is therefore essential to understand the diverse and evolving factors that influence the manifestation of aggressive behaviors during adolescence.

Notably, while signs of growing adolescent distress were already evident prior to the COVID-19 pandemic (Gunnell et al., 2018; McGorry and Mei, 2018), the pandemic exacerbated these trends, contributing to a marked rise in psychological malaise among youth (Close et al., 2024; Delaney, 2024; Foster et al., 2024; Jones et al., 2021; Singh et al., 2020). This raises pressing questions: How do adolescents navigate and integrate the aggressive impulses that are intrinsic to growth within the framework of adaptive, or maladaptive, needs? What factors facilitate aggressive expression in youth, at times manifesting as destructive or violent behavior?

The present work seeks to examine the contemporary role played by aggression in adolescence and explore its multifaceted expression.

## 2 Aggression during development

Aggression plays a fundamental role in human development, initially serving as a mechanism for survival and gradually evolving to support more adaptive and sophisticated behaviors. For instance, a newborn expresses discomfort and need through crying and protest. However, over time, the infant learns to tolerate short delays and begins to adopt more socially adaptive strategies (e.g., smiling, modulating vocalizations) to elicit care and satisfaction from the environment, and particularly caregivers (Winnicott, 2010, 2013). In this way, the child begins to understand that withholding immediate protest can result in more favorable outcomes.

This early modulation of aggressive impulses fosters the capacity to endure frustration and contributes to the development of emotional regulation. Aggression, intertwined with the experience of pleasure, helps form the first emotional bonds, initiates exploration, and supports psychological well-being, even at the cost of occasional self-restraint. As development progresses, the child learns to channel aggressive impulses into more evolved behaviors. These impulses may be inhibited, redirected, transformed, or projected, for example, through play, fantasy, or identification with aggressive figures such as superheroes, monsters, or characters in films and stories. However, the relatively balanced adaptation achieved in childhood is profoundly challenged during adolescence–a stage that reactivates and reorganizes previous developmental acquisitions rather than negating them entirely. This is a necessary reevaluation prompted by the transformative processes of growth (Sawyer et al., 2018).

During adolescence, biological drives, particularly those linked to aggression (e.g., conquest, possession, assertion) and sexuality, intensify, creating new internal needs that seek unfamiliar modes of fulfillment (Friedman, 1992). However, the adolescent is not yet neuropsychologically equipped to manage these increased internal and external demands.

Neurobiologically, this developmental phase is marked by significant changes in brain structure, involving both white and gray matter. The limbic system, which governs emotional responses, behavioral reactivity, and learning, matures earlier than the prefrontal cortex, responsible for executive functioning, planning, and impulse control. This temporal mismatch often results in reduced inhibition and heightened perceptual intensity, which may drive adolescents toward novel and risk-laden experiences (Holmes et al., 2016). Furthermore, the surge in sex hormones during puberty, exerting a significant effect on the limbic system, amplifies the adolescent’s pursuit of emotionally charged and stimulating experiences (Bos et al., 2018; Riedel et al., 2019; Romeo, 2017).

Thus, the disturbances and aggressive manifestations observed in adolescence can be understood as outward signs of the internal reorganization and adaptive recalibration underway during this complex period of growth.

### 2.1 The pleasure principle and the reality principle

Parents hold a fundamental responsibility in guiding their children’s development and, in particular, helping them navigate the tension between the pleasure principle and the reality principle. In early childhood, the child instinctively seeks to satisfy needs and desires, often with intensity and aggressiveness, expressing the pleasure principle (Klein, 1932). When these impulses are thwarted by parental limits, the child may initially respond with tantrums, disobedience, or emotional outbursts (Thompson, 1994). These reactions, while challenging, reflect a vital capacity for self-assertion.

Over time, the child begins to accept limits and renounce immediate gratification in favor of maintaining parental love and approval (Ainsworth et al., 1978; Bowlby, 1969). The rules and boundaries imposed by caregivers mark the child’s first encounter with external constraints, essentially, the shaping of reality. Parental intervention thus introduces the child to the reality principle, offering protection from danger, instilling respect for others’ property, and reinforcing core educational and social norms (Calkins, 2007; Kopp, 1982).

Through this process, the child learns to modulate behavior in accordance with reality. The pleasure principle, which dominates early development, becomes increasingly confined to internal fantasies. Its unregulated pursuit, marked by impulsive aggression, disregard for context, and lack of responsibility, must be reconciled with the demands of reality, which require the postponement of gratification and the regulation of aggressive impulses in service of personal affirmation within socially acceptable limits.

Children, by imitating their caregivers, figures who provide safety, affection, and stability, gradually internalize behavioral norms (Vygotsky, 1987). This process underpins the development of self-control, including the modulation of aggression, as a means of securing love and admiration from those they idealize (Bandura, 1977). The admired qualities of parents eventually inform broader social ideals, shaping the moral conscience. This internalized ethical structure governs the regulation of desire and impulse, rewarding conformity with self-esteem and punishing transgression with guilt or feelings of inadequacy (Kochanska, 2002).

Parenting style plays a crucial role in this developmental trajectory. Authoritarian or excessively permissive parenting (Masud et al., 2019), maternal overprotection (Zhang et al., 2022), and educational models rooted in social marginalization or disadvantage (Fauzi et al., 2023) have been associated with increased risk of maladaptive, aggressive behaviors. Family communication patterns, in particular, have been identified as among the most significant protective factors against emotional distress and deviant behavior in adolescence (Goering and Mrug, 2021; Mastronardi, 2002).

### 2.2 Aggressiveness in adolescence: a destructive or vital force?

In contemporary scientific discourse, aggression in adolescence is often categorized into two distinct forms: reactive aggression and proactive aggression (Hubbard et al., 2010; Polman et al., 2007; Romero-Martínez et al., 2022; Smeets et al., 2017). Reactive aggression is typically an impulsive, emotionally charged response to perceived provocation or threat, characterized by emotional dysregulation and heightened reactivity. In contrast, proactive aggression is premeditated and goal-oriented, an emotionally detached behavior enacted in the absence of provocation, often aimed at achieving a specific benefit or advantage.

These two forms of aggression may coexist within individuals, and several models recognize their complementary expression in different contexts. However, much of the current literature remains focused on examining aggression in relation to specific subpopulations or external variables, often at the expense of exploring the deeper, intrapsychic dimensions of aggressive behavior as it relates to an individual’s developmental trajectory. A more comprehensive understanding of adolescent aggressiveness requires an expanded conceptual lens.

Aggression may be broadly defined as a latent disposition, present in both thought and action, that can be expressed across a continuum, ranging from subtle hostility to overt acts of physical or psychological violence. At its extreme, such aggression may aim at the symbolic or literal annihilation of the other, reducing the other to a mere object to be dominated, harmed, or erased, devoid of subjectivity or human value. When aggressive behavior is driven by a narcissistic imperative, in which the negation of the other is central to self-gratification, it takes on a destructively dominant form. This corresponds to pleasure principle, wherein aggression is used unreservedly to achieve immediate satisfaction, without regard for social norms or the humanity of others.

In contrast, another conceptualization of aggression draws on the etymology of the Latin term adgredior, meaning “to approach, begin, or undertake” (Castiglioni and Mariotti, 2019). This framing imbues aggression with constructive and adaptive potential, emphasizing its role in personal assertion, initiative, and engagement with others. Under this view, aggression reflects a drive toward relationship, autonomy, and goal-directed behavior, aligning with the reality principle–the capacity to delay gratification and regulate impulses in harmony with environmental demands. From this perspective, prosocial behaviors such as helping, sharing, and cooperating are not antithetical to aggression, but may in fact represent its evolved, socially integrated forms. These behaviors have been identified as strong predictors of positive interpersonal relationships and healthy self-affirmation during adolescence (Arbel et al., 2022, 2023).

Although the two conceptualizations differ sharply in their orientation, destructive versus adaptive, they share a common foundation: aggression as an energy aimed at conquest, goal attainment, and the pursuit of personal agency.

It is thus possible to understand how aggression in adolescence can constitute a component of development and represent one of the growth languages (Winnicott, 1965). Aggression is nourished by the perception of an internal discomfort that the adolescent is not yet able to recognize or symbolize, and which is therefore expressed through behavior (Fonagy et al., 2002). In adolescence, new emotions, desires, and aspirations accompany the maturation and development of the body, modifying the relationship between the adolescent and the surrounding world (Erikson, 1968). The integration of such profound biological changes, psychological modifications, and different social rules is not easy (Steinberg, 2008). The process demands emotional sensitivity, cognitive processes, and impulsivity regulation, at a stage when the emotional-motivational complex is more reactive than cognitive control systems, which have not yet matured (Casey et al., 2008).

Adolescence is a period characterized by continuous testing of limits, self-comparison, relational experimentation, and the pursuit of autonomy. It is a stage that demands the forging of new internal and external equilibria, often exposing underlying vulnerabilities or narcissistic defenses. The adolescent experience, therefore, can be seen as a dynamic interplay, a “game of forces,” between the pleasure principle and the reality principle, each competing to shape the individual’s evolving identity and mode of being in the world.

**FIGURE 1:**
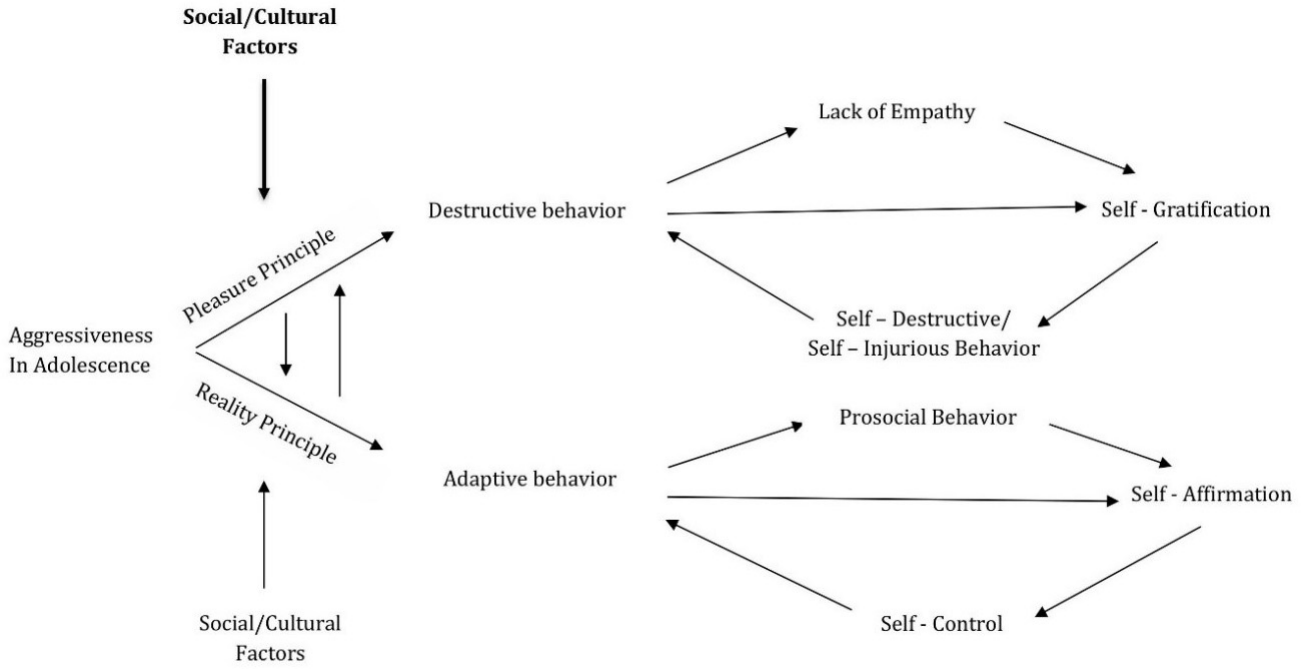
The balance of aggression in adolescence.

Adolescent aggression may manifest as a destructive force, driven by the pursuit of personal gratification without regard for others, in alignment with the pleasure principle, or as a vital, adaptive force of self-affirmation that respects moral conscience and social norms, consistent with the reality principle. The dominance of one form over the other is crucial in shaping the adolescent’s developmental trajectory, with implications for balanced and coherent identity formation (see Figure 1).

Aggression thus represents a broad phenomenon, including both vulnerability and disquiet, as well as the need for recognition and affirmation. For adolescents, it offers a way of managing overwhelming emotions, asserting one’s identity, and feeling strong.

In navigating this developmental balance, the emergence of new cultural paradigms plays a significant role (Kinghorn et al., 2018). Contemporary adolescents are increasingly being influenced by shifting cultural reference points and evolving behavioral models, often shaped by digital technologies.

The young person who begins to separate from their parents may develop their own “identity” that is not accessible to their parents, through a blog and/or presence on social media. However, this connection to technology may nourish their “social” self at the expense of their individuality (Dienlin and Johannes, 2020). Not feeling any state of mind when faced with the “machine,” the adolescent may be led to transform their image or acquire a “virtual identity” in an attempt to affirm, or rather “anesthetize” their presence in the world (Le Breton, 2002).

Lachance (2012) spoke of the peculiar relationship that “hypermodern adolescents” have with temporality through the conspicuous and ritual use of new technologies. Indeed, for adolescents (as with others), digital technologies change and induce a new relationship with time. In a society of uncertain contours, where true independence is often delayed, temporality may be grasped by young people as an attempt at emancipation.

These paradigms can undermine healthy identity construction by promoting a homogenization of experiences, valuing aesthetic ideals over individuality, and emphasizing personal success at the expense of relational and emotional depth (Choukas-Bradley et al., 2022).

Digital culture, in particular, perpetuates a culture of appearance and competitive individualism, often reinforcing aggressive tendencies. It fosters inequality, encourages confrontation, and hampers the development of emotional intelligence. Moreover, the misuse of digital platforms, through privacy violations or cyberbullying, has emerged as a widespread social issue (Caceres and Holley, 2023; Segura et al., 2020). Adolescents are constantly exposed to media saturated with violence, in which even heroic figures resort to aggression as a primary means of restoring order or asserting justice.

One notable psychological characteristic in aggressive and violent adolescents is a deficit in empathy. Empathy, which rests on self-awareness and emotional receptivity, is foundational to the reality principle and social functioning (Dorris et al., 2022). Adolescents with limited empathic capacity or callous-unemotional traits lack both the cognitive and the affective capacity to process the emotional states of others, thereby increasing the likelihood of aggressive behavior.

Goering and Mrug (2021) found that authoritative parenting (understood as open communication between parent and child) can promote empathy in early adolescence, and this increased empathy can reduce the likelihood of delinquency in late adolescence. Indeed, clear and supportive parenting helps adolescents develop better emotional skills and empathy, thereby disrupting dysfunctional and antisocial behavior.

Segura et al. (2020) found that empathy and emotional intelligence played a significant role in reducing cyber-aggressive behaviors and promoting positive relationships among adolescents. Thus, the promotion of empathy may be considered essential for preventing destructive aggression and encouraging the emergence of prosocial behaviors.

Children and adolescents’ ability to develop normative beliefs about aggression (Huesmann and Guerra, 1997) has a direct influence on their behavior–determining the extent to which they engage in aggressive behavior. Typically, this acquisition occurs through family experiences, peer relationships, and observed social patterns (including those observed on digital technology/media). Bailey and Ostrov (2008) analyzed the relationship between normative beliefs about aggression and the desire for social affirmation, finding that individuals with high social status often use acute forms of aggression to maintain power and control in their peer groups.

Adolescents’ brains are not yet mature (Blakemore and Robbins, 2012), and their decision-making tends to prioritize emotions and rewards (due to the early development of the socioemotional system characterized by the amygdala and ventral striatum) over cognitive control (due to the slower development of the prefrontal cortex, with implications for planning and consequence assessment). Children and adolescents learn to behave prosocially through rewards mechanisms, and these mechanisms change with age. While in children, prosociality is encouraged by external rewards (e.g., approval, praise), in adolescence, sensitivity to social rewards (i.e., peer acceptance, status) and the perception of personal satisfaction gains precedence. As adolescents develop, these circuits eventually integrate with areas of mentalization and empathy (i.e., medial prefrontal cortex, temporoparietal cortex) (Kwak and Huettel, 2016).

At times, this aggression may turn inward, manifesting in self-destructive or self-injurious behaviors, such as demeaning self-talk or acts of self-harm (Gillies et al., 2018). These behaviors reflect profound internal conflict and may signal borderline or psychopathological conditions, or broader patterns of maladaptation and antisociality (Beaudoin and Beauchamp, 2020; Bove et al., 2017; Ramírez and Andreu, 2006).

Sociocultural factors such as ethnicity, religion, and social stigma can also influence adolescent aggression. Often, these variables intersect with experiences of discrimination or marginalization, thereby further complicating behavioral outcomes (Goldston et al., 2008; Smokowski et al., 2017).

## 3 Conclusion

Adolescents are inherently vulnerable and developmentally fragile, with heightened susceptibility to aggressive behaviors and expressions (Martin et al., 2014; Sawyer et al., 2012). This work has aimed to explore the role that aggressiveness plays in contemporary adolescence and to understand the forms in which it manifests.

It may be posited that the dominance of either a self-referential, potentially alienating form of aggression or a prosocial, goal-directed aggression is critical in determining whether an adolescent’s developmental path is balanced and coherent. Both environmental influences and an individual’s personal history of development are key factors in shaping the trajectory and expression of these divergent forms of aggression.

A central paradox of contemporary society is its simultaneous promotion of individual supremacy and success, and aggressive behaviors that are narcissistically driven and lacking in empathy. Such behaviors are often legitimized and socially rewarded, thus becoming internalized by adolescents as part of a new, distorted “adaptive” reality principle. In such a context, destructive conduct and immediate gratification are valorized, undermining the adolescent’s capacity to channel aggression into more elaborate, respectful, and morally grounded forms of self-assertion.

As a result, adolescents may experience deep uncertainty in their expression of aggression as a healthy component of growth and identity formation. These dynamics underscore the need for multi-level interventions, recognizing that the educational and developmental responsibility for adolescents must be shared between the family, social institutions, and broader societal structures.

Investing in the recognition, understanding, and regulation of adolescent aggression may facilitate the emergence of more balanced behaviors and support the development of meaningful interpersonal relationships fostering personal growth and psychological well-being. Ultimately, we must understand adolescent aggression as a potent expression of self-affirmation, a complex, often ambivalent force that is intrinsic to the human experience of growing up.

## Data Availability

The original contributions presented in this study are included in this article/supplementary material, further inquiries can be directed to the corresponding author.
